# Effects of Glyphosate-Based Herbicide on Primary Production and Physiological Fitness of the Macroalgae *Ulva lactuca*

**DOI:** 10.3390/toxics10080430

**Published:** 2022-07-28

**Authors:** Ricardo Cruz de Carvalho, Eduardo Feijão, Ana Rita Matos, Maria Teresa Cabrita, Andrei B. Utkin, Sara C. Novais, Marco F. L. Lemos, Isabel Caçador, João Carlos Marques, Patrick Reis-Santos, Vanessa F. Fonseca, Bernardo Duarte

**Affiliations:** 1 MARE—Marine and Environmental Sciences Centre & ARNET—Aquatic Research Infrastructure Network Associated Laboratory, Faculdade de Ciências, Universidade de Lisboa, Campo Grande, 1749-016 Lisboa, Portugal; emfeijao@fc.ul.pt (E.F.); micacador@fc.ul.pt (I.C.); vffonseca@fc.ul.pt (V.F.F.); baduarte@fc.ul.pt (B.D.); 2 cE3c—Centre for Ecology, Evolution and Environmental Changes, Faculdade de Ciências, Universidade de Lisboa, Campo Grande, Edifício C2, Piso 5, 1749-016 Lisboa, Portugal; 3 Departamento de Biologia Vegetal, Faculdade de Ciências, Universidade de Lisboa, Campo Grande, 1749-016 Lisboa, Portugal; armatos@fc.ul.pt; 4 BioISI—Biosystems and Integrative Sciences Institute, Faculdade de Ciências, Universidade de Lisboa, 1749-016 Lisboa, Portugal; 5 Centro de Estudos Geográficos (CEG), Instituto de Geografia e Ordenamento do Território (IGOT), Universidade de Lisboa, Rua Branca Edmée Marques, 1600-276 Lisboa, Portugal; tcabrita@campus.ul.pt; 6 Laboratório Associado Terra, Instituto Superior de Agronomia, Universidade de Lisboa, Tapada da Ajuda, 1349-017 Lisboa, Portugal; 7 INOV-INESC, Rua Alves Redol 9, 1000-029 Lisboa, Portugal; andrei.utkin@inov.pt; 8 CeFEMA, Universidade de Lisboa, Av. Rovisco Pais 1, 1049-001 Lisboa, Portugal; 9 MARE—Marine and Environmental Sciences Centre & ARNET—Aquatic Research Infrastructure Network Associated Laboratory, Department of Life Sciences, ESTM, Politécnico de Leiria, 2520-630 Peniche, Portugal; sara.novais@ipleiria.pt (S.C.N.); marco.lemos@ipleiria.pt (M.F.L.L.); 10 MARE—Marine and Environmental Sciences Centre & ARNET—Aquatic Research Infrastructure Network Associated Laboratory, Department of Life Sciences, Faculty of Sciences and Technology, University of Coimbra, 3000-071 Coimbra, Portugal; jcmimar@ci.uc.pt; 11 Southern Seas Ecology Laboratories, School of Biological Sciences, The University of Adelaide, Adelaide, SA 5005, Australia; patrick.santos@adelaide.edu.au; 12 Departamento de Biologia Animal, Faculdade de Ciências, Universidade de Lisboa, Campo Grande, 1749-016 Lisboa, Portugal

**Keywords:** photobiology, energetic metabolism, pesticide, oxidative stress, glyphosate

## Abstract

The use of glyphosate-based herbicides (GBHs) worldwide has increased exponentially over the last two decades increasing the environmental risk to marine and coastal habitats. The present study investigated the effects of GBHs at environmentally relevant concentrations (0, 10, 50, 100, 250, and 500 μg·L^−1^) on the physiology and biochemistry (photosynthesis, pigment, and lipid composition, antioxidative systems and energy balance) of *Ulva lactuca*, a cosmopolitan marine macroalgae species. Although GBHs cause deleterious effects such as the inhibition of photosynthetic activity, particularly at 250 μg·L^−1^, due to the impairment of the electron transport in the chloroplasts, these changes are almost completely reverted at the highest concentration (500 μg·L^−1^). This could be related to the induction of tolerance mechanisms at a certain threshold or tipping point. While no changes occurred in the energy balance, an increase in the pigment antheraxanthin is observed jointly with an increase in ascorbate peroxidase activity. These mechanisms might have contributed to protecting thylakoids against excess radiation and the increase in reactive oxygen species, associated with stress conditions, as no increase in lipid peroxidation products was observed. Furthermore, changes in the fatty acids profile, usually attributed to the induction of plant stress response mechanisms, demonstrated the high resilience of this macroalgae. Notably, the application of bio-optical tools in ecotoxicology, such as pulse amplitude modulated (PAM) fluorometry and laser-induced fluorescence (LIF), allowed separation of the control samples and those treated by GBHs in different concentrations with a high degree of accuracy, with PAM more accurate in identifying the different treatments.

## 1. Introduction

The marine environment is subjected to increasing levels of pollution [1]. The exponential speed of innovation in synthetic chemicals (25 million in 2005 up to 100 million in 2015) [2] has led to serious concerns about the ecotoxicity of emerging pollutants and the efficiency of monitoring methodologies [3]. Emerging pollutants include pesticides, pharmaceuticals, and personal and household care products, which are used daily worldwide [4].

Glyphosate (N-(phosphonomethyl) glycine) is a broad-spectrum herbicide, acting as a glycine analog, which inhibits the enzyme 5-enolpyruvyl-shikimate-3-phosphate synthase (EPSPS) of the shikimate pathway, affecting the aromatic amino acid biosynthesis pathway [5]. The effect of glyphosate on the activity of EPSPS also produces indirect effects in photosynthesis causing inhibition of carbon assimilation, depletion of intermediates of the photosynthetic carbon reduction cycle due to deregulation of the shikimate pathway, alterations in the concentrations of carotenoids, chlorophyll *a*, fatty acids, and proteins in the reaction centers of photosystem II (PSII) [6,7,8,9,10,11,12,13].

Glyphosate is the main active ingredient in the commercial mixture Roundup^®^ herbicide that has been used worldwide for over 40 years [14,15]. Over 20 years (1994–2014), glyphosate-based herbicide (GBH) use increased from 56 million to more than 826 million kg globally, associated with agricultural and non-agricultural practices [16]. This rise is also associated with the introduction of glyphosate-tolerant crops, which possess either the tolerant EPSPS synthase gene, a glyphosate metabolism gene or both genes [17]. After application, GBHs can reach water bodies from a direct application or indirect transport by wind and/or runoff after intense rainfalls [18,19]. Although glyphosate is not bound to the soil, it can be metabolized by microbes into aminomethylphosphate and CO_2_ [20] and, part of it is adsorbed onto the soil, decreasing its biodegradation, and increasing its persistence time [21]. Furthermore, the half-life of glyphosate in soil ranges from 2 to 197 days [22], and in water ranges from 3 to 91 days [23]. In vitro experiments showed that the half-life for glyphosate in seawater ranged from 47 days (in low light) up to 315 days (in the dark) [24].

The toxicity of GBH formulations has been investigated in several aquatic organisms (e.g., [13,25,26,27,28,29]. However, investigations of its effects on macroalgae are scarcer [30,31,32,33]. Changes in marine microbial communities were associated with GBH application [26] affecting, for example, cyanobacteria and diatoms at low concentrations (50 µg·L^−1^) [13,34], while in macroalgae, deleterious effects were observed at higher concentrations (176.5 µg·L^−1^) [31].

Runoff from agriculture and sewage effluents increase the amount of pollution in estuarine, coastal, and marine water bodies, reaching and affecting all living organisms, from primary producers to animals [35,36]. Guidelines to protect biodiversity and the functioning of estuarine, coastal, and marine environments need to be established through ecosystem-level ecotoxicological studies [37,38,39]. Macroalgae (or seaweeds) are a very relevant portion of the primary producers and an important component of the trophic web as part of the diet of fish and many other animal species [40], containing high amounts of carbohydrates (up to 60%), medium/high amounts of proteins (10–47%) and low amounts of lipids (1–3%) [41]. *Ulva lactuca* (Ulvaceae, Chlorophyta), commonly known as sea lettuce, is a macroalga that can be found in marine and estuarine environments being ubiquitous in coastal benthic communities around the world [42,43]. This species can undergo a sudden and uncontrolled proliferation in response to environmental stress, such as climate change or water eutrophication [44], covering the water surface and decreasing biodiversity, even for other algae species [45]. Therefore, *Ulva* species are good candidates for ecotoxicology studies leading to the development of many bioassays, such as the UlvaTox bioassay which have been used in the biomonitoring of metals (e.g., silver, arsenic, cadmium, cobalt, chromium, copper, iron, mercury, manganese, nickel, lead, and zinc), formalin, diesel fuel, tributyltin oxide, and thiazolidinediones in municipal wastewater and industrial effluents [46,47,48,49,50,51]. The cell wall of *U. lactuca* is composed of a highly charged mucopolysaccharide, named ulvan, allowing the adsorption of those pollutants, such as GBHs [44,52]. Although this mechanism has been suggested for bioremediation and biomonitoring [52,53], the effects on *U. lactuca* physiology and biochemistry are less known.

Therefore, the use of non-invasive high-throughput screening tools, such as pulse amplitude modulated (PAM) fluorometry and laser-induced fluorescence (LIF), allows evaluation of the ecotoxicity in photosynthetic organisms [53,54,55,56,57]. While PAM has been widely used as a proxy for the bioenergetics involved in photosynthesis and the physiological effects of different concentrations of contaminants [13,55,58,59,60] and was previously used to study macroalgae [61], LIF has seen a greater development over the last decade [62,63] and previously used in macroalgae including *Ulva* [56]. In green leaves, presenting a maximum in the red region (Fr) and at the far-red (Ffr) region of the spectrum, LIF spectra and the Fr/Ffr ratio have a good correlation with the maximum photochemical efficiency of PSII (F_v_/F_m_) and with chlorophyll *a* concentration, with clear deviations under stress conditions [64].

Although studies have targeted GBH effects on diatoms, the fact that diatoms are single-cell organisms presents some limitations regarding long-term effects and extrapolations to more complex autotrophs. Macroalgae, having differentiated tissues, may serve as a model for marine autotrophic organisms with higher complexity. Since glyphosate can persist in water from weeks to several months [23] and macroalgae have successfully been used as pollution biomonitors [65], this study aimed to assess the ecotoxicological effects of GBH exposure to concentrations currently found in estuarine and marine ecosystems [66,67,68,69]. The photosynthesis, antioxidative systems, pigment, fatty acid composition, and energy balance of the cosmopolitan macroalga *Ulva lactuca* were analyzed, identifying the best biomarkers for GBH contamination and its potential effects.

## 2. Material and Methods

### 2.1. Experimental Setup

The macroalgae *Ulva lactuca* was acquired from AlgaPlus (Ílhavo, Portugal) and maintained for one week in culture before the assay. The culture medium consisted of filtered seawater enhanced with Provasoli’s Enriched Medium (PES medium) [70]. The macroalgae were kept in an aquarium under controlled conditions (18 ± 1 °C, under constant aeration and a 14 h light: 10 h dark photoperiod). The growth chamber was programmed to simulate sunrise and sunset using a sinusoidal function with a light intensity at noon simulating a natural light environment (RGB 1:1:1, Maximum PAR 80 μmol photons m^−2^ s^−1^, 14/10 h day/night rhythm). For the assay, disks with a 1.5 cm diameter were cut and introduced into plastic plates with twenty-four wells of the same diameter. The disks were exposed to 0, 10, 50, 100, 250, or 500 μg·L^−1^ solution of glyphosate-based herbicide (GBH) for 48 h [13], being prepared from the GBH “Roundup^®^ Pronto” containing 7.2 g L^−1^ glyphosate. These concentrations were chosen based on the current literature regarding the range of environmental concentrations found in agricultural water streams [66,67,68,69]. For each herbicide concentration, 30 replicates were made. To avoid contamination, all labware was washed with HNO_3_ (20%) for two days, rinsed thoroughly with ultra-pure water and autoclaved. All culture manipulations were performed in a laminar airflow chamber using aseptic techniques.

### 2.2. Chlorophyll a Pulse Amplitude Modulated (PAM) Fluorometry

Chlorophyll fluorescence measurements were performed through pulse amplitude modulated fluorometry using a FluoroPen FP100 (Photo System Instruments, Drásov, Czech Republic), on 15 min dark-adapted samples. Analysis of chlorophyll transient light curves (OJIP curves or Kautsky plots) was conducted using the OJIP test [71]. All fluorometric parameters and their description can be accessed in Supplementary Materials (Table S1).

### 2.3. Laser-Induced Fluorescence (LIF) Analysis

The fluorescence emission spectra of *Ulva lactuca* disks were obtained following the procedure described in [56] using a LIF sensor built around a frequency-doubled Nd:YAG laser (Quantel, model Ultra 532 30 20HN, Newbery, UK) and a custom low-noise photodetector described previously in [64,72].

The laser emitter produced 532 nm wavelength radiation pulses (duration of 5 ns and energy of 12 mJ) at a 5 Hz rate, the single pulse fluence at the target being <7.5 mJ cm^−2^. The receiver (fluorescence-emission gathering optics) was developed using the Thorlabs optomechanical elements within the one-inch-diameter lens-mounting tube SM1L30. It contained the longpass optical filter FEL0550, with a cut-off wavelength of 550 nm and transmission of ~80% for the 650–730 nm spectral range, protecting the sensitive optoelectronics from strong laser light at 532 nm. The light-gathering optics was connected to the photodetector through a custom-made optical fiber provided by Thorlabs (365 µm core, 0.22 numerical aperture), providing the best collimator-fiber-spectrometer optical matching. The photodetector was based on a commercial spectrometer from Ocean Optics having the f/4 asymmetrical crossed CzernyTurner optical bench. The widest possible (for the given optomechanical system) entrance slit, 0.2 × 1 mm^2^, was installed to provide maximum sensitivity, and an additional cylindrical lens was placed in front of the detector window to focus the light on the photodetection array (Toshiba TCD1304AP).

### 2.4. Pigment Analysis

Pigment extraction was performed according to previous works [53,57,73]. After samples were freeze-dried for 48 h, pure acetone was added, and extraction occurred at −20 °C for 24 h to prevent degradation. Samples were centrifuged for 15 min at 4000× *g* and 4 °C (Sigma 2–16K, Sigma Laborzentrifugen GmbH, Landkreis Osterode, Germany) and the supernatants were scanned by a dual-beam spectrophotometer from 350 nm to 750 nm at 0.5 nm steps (Shimadzu UV-1603, Shimadzu Co., Kyoto, Japan). Using the SigmaPlot Software, the absorbance spectrum was introduced in the Gauss-Peak Spectra (GPS) fitting library. Pigment analysis was performed according to [74], allowing the detection of chlorophyll *a* and *b*, pheophytin *a* and *b*, lutein, β-Carotene, zeaxanthin, antheraxanthin, violaxanthin, and auroxanthin.

### 2.5. Antioxidant Enzyme Assays

After homogenization in mortar and pestle with liquid nitrogen, the soluble protein fraction was extracted at 4 °C in 500 µL of 50 mM sodium phosphate buffer (pH 7.6) with 0.1 mM Na-EDTA. The homogenate was centrifuged at 15,000× *g* for 10 min at 4 °C to remove debris, and the supernatant was collected into a new tube.

The enzyme activity measurements of ascorbate peroxidase, superoxide dismutase, catalase, and glutathione reductase were performed at 25 °C in a microplate reader spectrophotometer (Epoch 2 Microplate Reader, BioTek Instruments, Winooski, VT, USA). Ascorbate peroxidase (APX) was assayed according to [75]. Ascorbate oxidation was monitored as the decrease in absorbance at 290 nm (ε = 2.8 mM^−1^ cm^−1^). Superoxide dismutase (SOD) activity was assayed according to [76] by monitoring the reduction in pyrogallol and its increase in absorbance at 325 nm. The autoxidation of pyrogallol was read without enzymatic extract during the same time interval for comparison enabling. Catalase (CAT) activity was measured according to [77], monitoring H_2_O_2_ consumption and consequent decrease in absorbance at 240 nm (ε = 39.4 M^−1^ cm^−1^). Glutathione reductase (GR) activity was assayed according to [78] by monitoring glutathione-dependent oxidation of NADPH and its decrease in absorbance at 340 nm (ε = 6.22 mM^−1^ cm^−1^). Protein concentration was determined according to [79].

### 2.6. Lipid Peroxidation Analysis

Lipid peroxidation products were determined according to [80]. Sample disks were homogenized briefly in 1 mL of a freshly prepared solution containing 20% (*v*/*v*) Trichloroacetic acid (TCA) and 0.5% (*w*/*v*) Thiobarbituric acid (TBA) and placed in an ultra-sound bath for 1 min. The reaction was conducted at 90 °C for 30 min, immediately stopped in ice and after centrifugation at 15,000× *g* for 10 min at 4 °C, the absorbance at 532 nm and 600 nm of the supernatant was recorded by spectrophotometry (Shimadzu UV-1603, Shimadzu Co., Kyoto, Japan). The concentration of malondialdehyde (MDA) was determined using the molar extinction coefficient (ε = 155 mM^−1^ cm^−1^).

### 2.7. Fatty Acid Profiles

The fatty acid analysis was performed according to [73] by direct trans-esterification of samples, in freshly prepared methanol sulfuric acid (97.5:2.5, *v*/*v*), at 70 °C for 60 min, using the internal standard pentadecanoic acid (C15:0). Fatty acids methyl esters (FAMEs) were recovered using petroleum ether, dried with an N_2_ flow, and re-suspended in hexane. Through gas chromatography (Varian 430-GC gas chromatograph equipped with a hydrogen flame ionization detector set at 300 °C), 1 µL of the FAME solution was analyzed, setting the injector temperature to 270 °C, with a split ratio of 50. The fused-silica capillary column (50 m × 0.25 mm; WCOT Fused Silica, CP-Sil 88 for FAME; Varian) was maintained at a constant nitrogen flow of 2.0 mL min^−1^ and the oven was set to 190 °C. Fatty acid identification was performed by comparison of retention times with standards (Sigma-Aldrich, Darmstadt, Germany) and chromatograms analyzed by the peak surface method, using the Galaxy software. The internal standard used was the pentadecanoic acid (C15:0). To determine the membrane saturation levels, the double bond index (DBI) was calculated according to [73]:

$$DBI=\frac{2\times \left(\%\;\mathrm{monoenes}+2\times \%\;\mathrm{dienes}+3\times \%\;\mathrm{trienes}+4\times \%\;\mathrm{tetraenes}+5\times \%\;\mathrm{pentaenes}\right)}{100}$$

### 2.8. Energy Balance

The cellular energy allocation (CEA) was measured following the procedures described in [13] by integrating the energy available (Ea) using total lipid, carbohydrate, and protein content, with the energy consumption (Ec) by the measurement of the mitochondrial Electron Transport System (ETS) activity as a proxy of cellular oxygen consumption and metabolism. Briefly, sample disks were homogenized by mortar and pestle using 1 mL of Milli-Q water. Extraction and quantification of total lipids, proteins and carbohydrates were performed according to [81,82] with minor modifications [13,83] and transforming the results into energetic equivalents (combustion energies: 17,500 mJ mg carbohydrates^−1^, 24,000 mJ mg protein^−1^, and 39,500 mJ mg lipid^−1^) [84]. ETS was determined according to [85] with major modifications [13,83] and mitochondrial oxygen consumption was estimated based on the theoretical stoichiometric relationship that for each 2 μmol of INT-formazan formed, 1 μmol of O_2_ was consumed in the ETS. Oxygen consumption was then transformed into energetic equivalents by using the specific oxyenthalpic equivalents for an average lipid, protein, and carbohydrate mixture of 480 kJ mol O_2_^−1^ [84]. In all assays, milli-Q water was used as a reaction blank. The CEA was then calculated as follows [86]:

$$CEA=\frac{Ea}{Ec}$$

where

$$Ea\;\left(available\;energy\right)=carbohydrate+lipid+protein\;{\left({\mathrm{mJ}\;\mathrm{mg}}^{-1}\;fresh\;weight\right)}^{}$$

$$Ec\;\left(energy\;consumption\right)=ETS\;activity\;\left({\mathrm{mJ}\;\mathrm{mg}}^{-1}\;fresh\;weight\right)$$

### 2.9. Statistical Analysis

All Statistical analysis was performed with R Statistical Software version 4.1.2 (R Core Team, 2021) using RStudio version 2021.09.2+382 (RStudio Team, 2021). Since neither normality (Shapiro–Wilk test) nor homoscedasticity (regression residues) requirements were met, Kruskal–Wallis analysis of variance was performed, together with using the ‘agricolae’ package (version 1.3–5) [87]. Posthoc tests were performed using Fisher’s least significant difference criterion realized with Bonferroni correction. Linear discriminant analysis (LDA) was performed using the MASS package and applied to the OJIP and LIF raw datasets allowing the classification and separation into different treatment groups.

## 3. Results

### 3.1. Macroalgae Photochemistry

**Figure 1:**
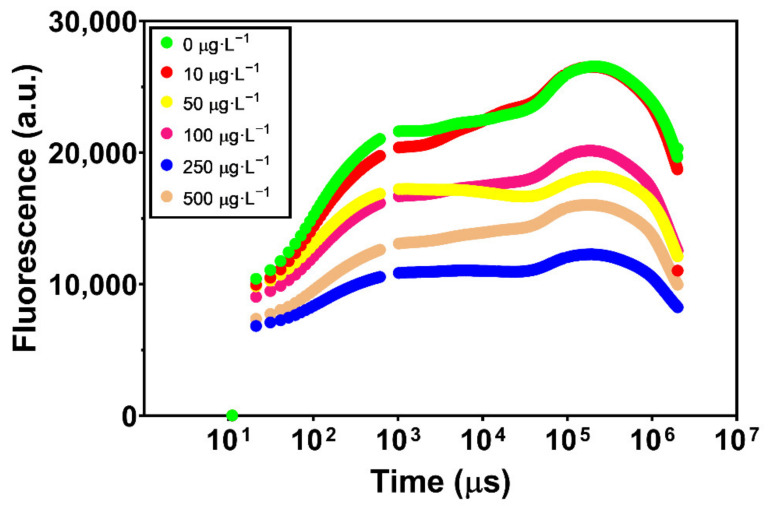
Chlorophyll transient kinetics (OJIP curves or Kautsky plots) in *Ulva lactuca* following a 48-h exposure to a glyphosate-based herbicide formulation in different concentrations (mean, *n* = 18).

The analysis of the chlorophyll transient kinetics (OJIP curves or Kautsky plots) showed that, apart from the lowest concentration (10 µg·L^−1^), fluorescence emission decreased with increasing exposure to GBHs (Figure 1), especially at the highest concentrations (250 and 500 µg·L^−1^), although 250 µg·L^−1^ showed the lowest fluorescence values.

**Figure 2:**
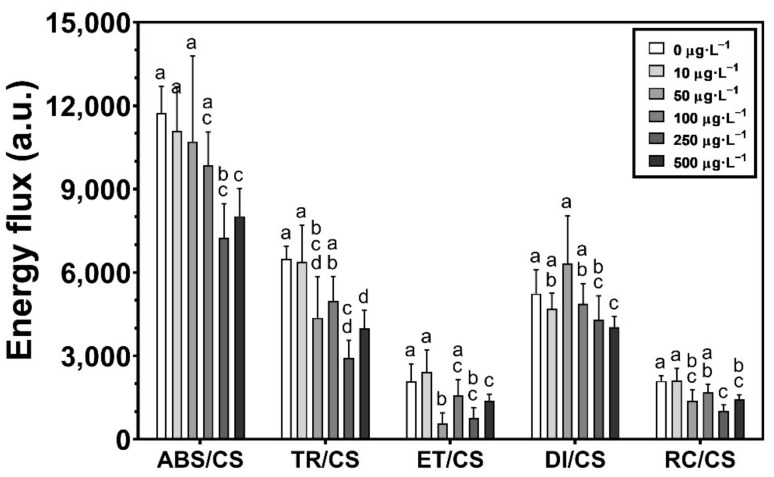
The energy fluxes (absorbed (ABS/CS, trapped (TR/CS), transported (ET/CS) and dissipated (DI/CS)) and the number of available reaction centers per cross-section (RC/CS), in *Ulva lactuca* following a 48-h exposure to a glyphosate-based herbicide formulation in different concentrations (mean ± s.d., *n* = 18, different letters indicate significant differences between treatments at *p* < 0.05).

After processing the previous curves, the effects of exposure to the different herbicide concentrations in the photochemical process from light-harvesting electronic transport are evident, particularly in the higher concentrations (250 and 500 µg·L^−1^), presenting decreasing values in the four main energy fluxes (Figure 2). An interesting fact is how the concentration of 50 µg·L^−1^ affects *U. lactuca*, presenting values similar to the two highest concentrations. Although the amount of energy absorbed by the PS II antennae (ABS/CS) with 50 µg·L^−1^ is similar to control, the energy flux that was effectively trapped inside the PS II (TR/CS) is significantly lower, also observed in the case of the energy transported within the electron transport chain (ET/CS). On the other hand, the energy dissipation flux (DI/CS) is significantly different from the two highest concentrations but the reduction of the number of oxidized PS II reaction centers (RC/CS) is similar in these three concentrations (50, 250, and 500 µg·L^−1^).

**Figure 3:**
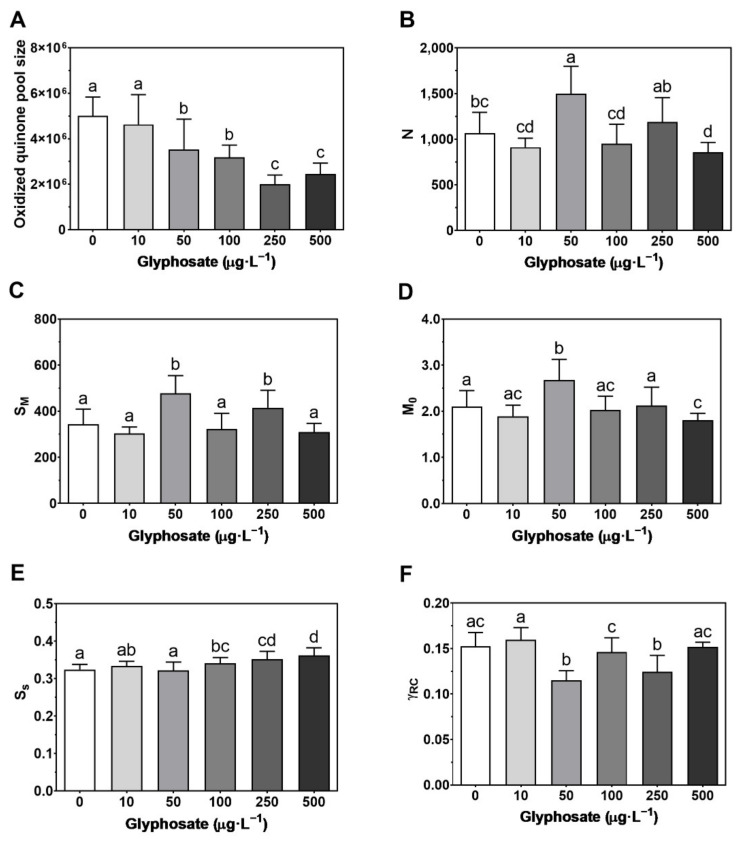
Photosystem II and ETC-related photochemical traits: (**A**) oxidized quinone pool; (**B**) reaction center turnover rate (N); (**C**) the energy needed to close all reaction centers (S_M_); (**D**) the net rate of PS II RC closure (M_0_); (**E**) smallest possible normalized total area when each QA is reduced only once (S_S_); (**F**) the probability that a PSII chlorophyll molecule function as an RC (γ_RC_), in *Ulva lactuca* following a 48-h exposure to a glyphosate-based herbicide formulation in different concentrations (mean ± s.d., *n* = 18, different letters indicate significant differences between treatments at *p* < 0.05).

Through a deeper analysis, the different components of the photosystems and the electron transport chain (ETC) in response to GBH concentration were determined (Figure 3). Regarding the oxidized quinone pool size (Figure 3A), there was a decrease between the 50 and 500 µg·L^−1^ concentrations, being distinguished into three groups: low (control and 10 µg·L^−1^), intermediate (50 and 100 µg·L^−1^) and high (250 and 500 µg·L^−1^) concentrations. However, only in the 50 µg·L^−1^ treatment was an enhancement in the number of QA redox turnovers until maximum fluorescence is reached (N) observed (Figure 3B), a pattern also observed in the energy needed to reduce all RCs (S_M_) (although this increase was also observed under 250 µg·L^−1^ (Figure 3C)) and in the QA reduction rate (M_0_) (Figure 3D). The highest concentration induced a decrease in both N and M_0_. Nevertheless, in the three highest GBH exposure concentrations (100, 250 and 500 µg·L^−1^), there was an increase in S_S_ (the smallest possible normalized total area when each QA is reduced only once, i.e., single turnover) (Figure 3E). At the 50 and 250 µg·L^−1^ concentrations, a decrease in the probability of a PS II chlorophyll molecule functioning as an RC (γ_RC_) was observed (Figure 3F).

**Figure 4:**
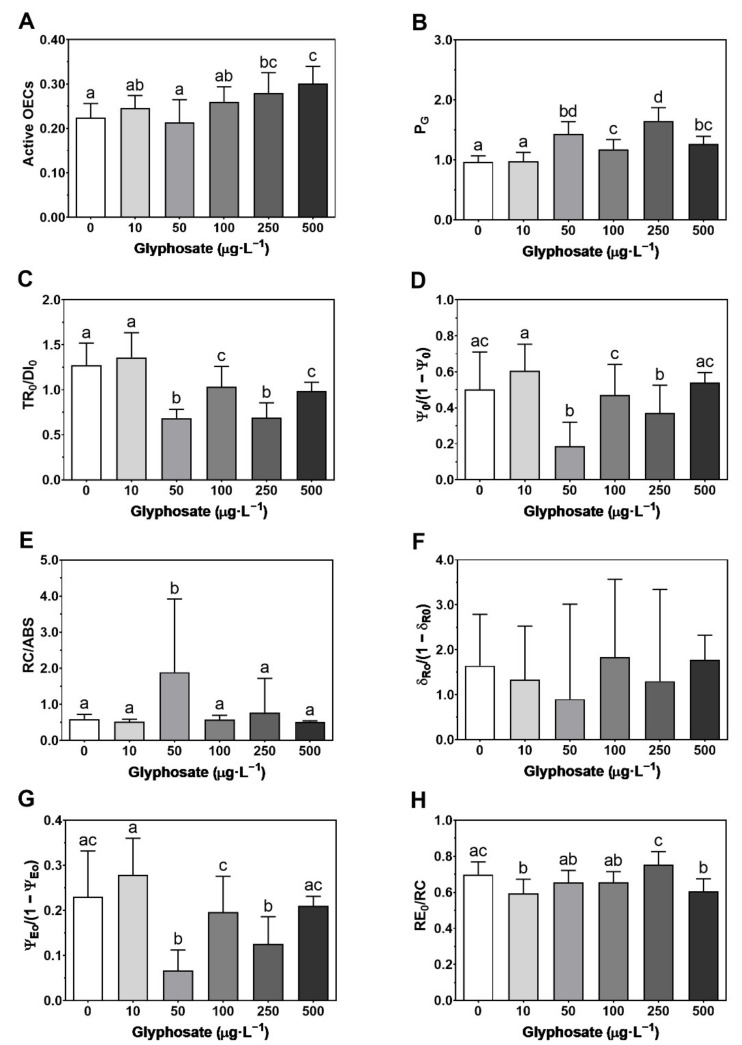
Photosystem II and PS I photochemical traits: (**A**) active oxygen-evolving complexes (OECs); (**B**) grouping probability between the two PSII units (P_G_); (**C**) contribution or partial performance due to the light reactions for primary photochemistry (TR_0_/DI_0_); (**D**) the contribution of the dark reactions from quinone A to plastoquinone (ψ_0_/(1 − ψ_0_)); (**E**) reaction center II density within the antenna chlorophyll bed of PS II (RC/ABS); (**F**) the contribution of PSI reducing its end acceptors (δ_R0_/(1 − δ_R0_); (**G**) the equilibrium constant for the redox reactions between PS II and PS I (ψ_E0_/(1 − ψ_E0_); (**H**) electron transport from PQH_2_ to the reduction of PS I end electron acceptors (RE_0_/RC)), in *Ulva lactuca* following a 48-h exposure to a glyphosate-based herbicide formulation in different concentrations (mean ± s.d., *n* = 18, different letters indicate significant differences between treatments at *p* < 0.05).

The active oxygen-evolving complexes (OECs) showed an increase, relative to the control, under the two highest GBH concentrations (250 and 500 µg·L^−1^) (Figure 4A), and an increase in the P_G_ (disconnection between the two PSII units) not only with the highest concentrations (250 and 500 µg·L^−1^) but also with the intermediate ones (50 and 100 µg·L^−1^) (Figure 4B). Photochemical processes between PS II and PS I showed a significant decrease in light (TR_0_/DI_0_) reactions of the photochemical cycle in concentrations ranging between 50 and 500 µg·L^−1^ (Figure 4C), but the decrease was only observed in 50 and 250 µg·L^−1^ concentrations for dark (ψ_0_/1 − ψ_0_) reactions of the photochemical cycle (Figure 4D). However, no significant changes were observed in the reaction center density within the PS II antenna chlorophyll bed (RC/ABS), apart from the increase at 50 µg·L^−1^ (Figure 4E), and in the activity, at the PS I level (δ_R0_/1 − δ_R0_) (Figure 4F). This led to a decrease in the equilibrium constant for the redox reaction between both photosystems towards the PS II (ψ_E0_/(1 − ψ_E0_)), in this case affecting not only the treatment with 50 µg·L^−1^ but also with 250 µg·L^−1^ (Figure 4G). Regarding the electron transport from PQH_2_ to the reduction in the PS I end acceptors (RE_0_/RC) (Figure 4H), the response was a decrease upon exposure with 10 and 500 µg·L^−1^, relatively to the control.

In the rapid light curve (RLC)-derived parameters, no significant differences were observed in the photosynthetic efficiency (α), the relative maximum electron transport rate (rETR_max_), or light saturation (E_k_), although there was a decrease in photoinhibition (β) when subjected to 100 µg·L^−1^ (Supplementary Figure S1).

### 3.2. Macroalgae LIF Analysis

**Figure 5:**
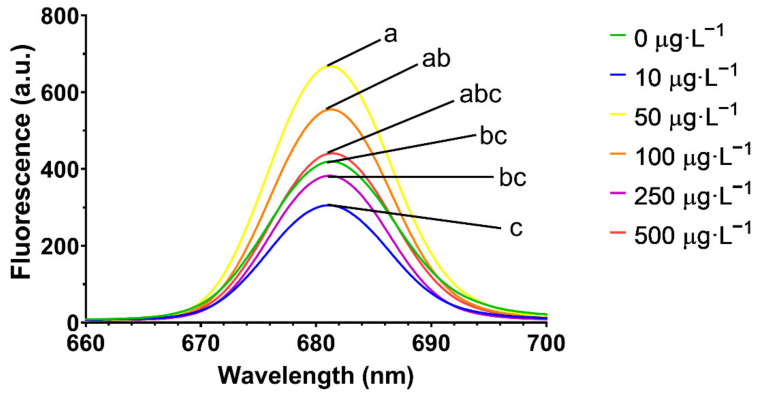
Red region laser-induced fluorescence in *Ulva lactuca* following a 48-h exposure to a glyphosate-based herbicide formulation in different concentrations (mean, *n* = 30, different letters indicate significant differences between treatments at *p* < 0.05).

Concerning the LIF analysis, *U. lactuca* presented a fluorescence peak around 681 nm (red region), with 50 µg·L^−1^ GBH exposed samples being the only ones to display a significant increase against the control (Figure 5).

On the other hand, while only the highest GBH concentration (500 µg·L^−1^) showed a significant increase in wavelength at maxima fluorescence in the red region relative to the control (Table 1), no changes were observed in the wavelength at maxima fluorescence in the far-red region. The red/far-red fluorescence ratios (F_680_/F_735_) showed a significant increase, compared to the control, in samples subjected to the GBH concentrations between 50 and 500 µg·L^−1^ (except for 100 µg·L^−1^).

### 3.3. Macroalgae Pigment Composition

Regarding the pigment composition, a decrease in both chlorophyll *a* and *b* was observed between 10 and 100 µg·L^−1^ GBH, returning to control values in the higher concentrations (250 and 500 µg·L^−1^) (Table 2). However, pheophytin *a* and *b* do not present any significant changes relative to the control. In the carotenoid-xanthophyll biosynthetic pathway, while there is a significant decrease in lutein at the lowest GBH dose (10 µg·L^−1^) it returns to control values at the highest concentration (500 µg·L^−1^), while changes in the remaining concentrations are not statistically significant. On the other hand, while β-carotene, zeaxanthin, auroxanthin and violaxanthin showed no changes relative to control, there was a significant accumulation of antheraxanthin at the highest GBH concentration (500 µg·L^−1^).

### 3.4. Macroalgae Antioxidant System

**Figure 6:**
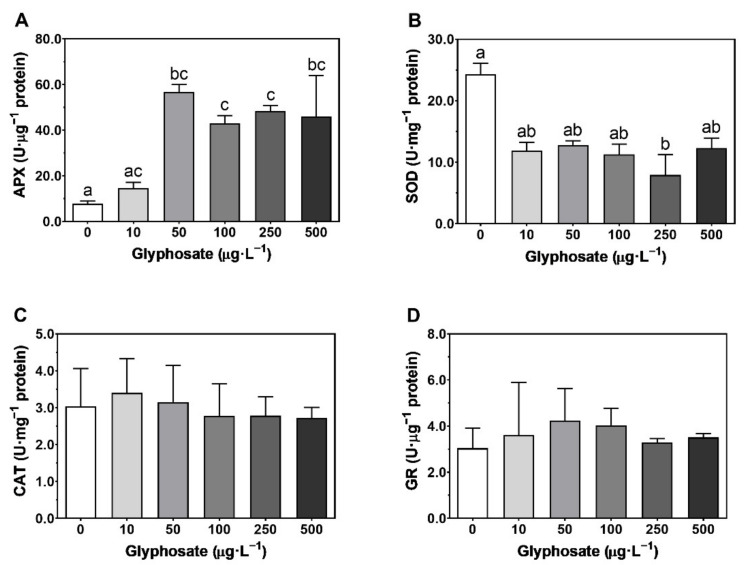
Ascorbate peroxidase (APX, (**A**)), superoxide dismutase (SOD, (**B**)), catalase (CAT, (**C**)), and glutathione reductase (GR, (**D**)) enzymatic activities in *Ulva lactuca* following a 48-h exposure to a glyphosate-based herbicide formulation in different concentrations (mean ± s.d., *n* = 3, different letters indicate significant differences at *p* < 0.05).

Regarding the antioxidant enzymes, APX showed higher activity in the concentrations between 50 and 500 µg·L^−1^ (Figure 6A) while SOD only showed significantly lower activity at 250 µg·L^−1^ relative to the control (Figure 6B). CAT and GR showed no statistical differences between treatments (Figure 6C,D).

Furthermore, no statistical differences were found in the lipid peroxidation (Supplementary Figure S2) of *U. lactuca* between treatments of GBH concentrations.

### 3.5. Macroalgae Fatty Acid Profile

**Figure 7:**
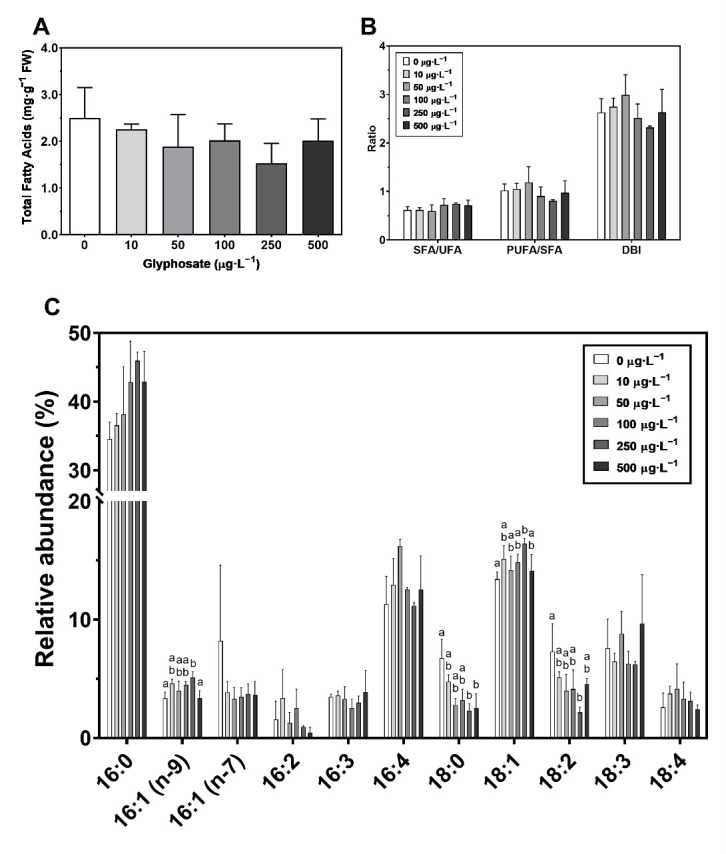
Total fatty acid content: (**A**) fatty acid ratios (saturated to unsaturated fatty acids ratio [SFA/UFA], polyunsaturated to saturated fatty acids ratio [PUFA/SFA] and double-bound index [DBI]); (**B**) and fatty acid relative abundance profile (%); (**C**) in *Ulva lactuca* following a 48-h exposure to a glyphosate-based herbicide formulation in different concentrations (mean ± s.d., *n* = 3, different letters indicate significant differences at *p* < 0.05).

Although variations in the total fatty acids content were not significant, there is a trend for a slight decrease in total lipids (Figure 7A), on a fresh weight basis, affecting mainly unsaturated fatty acids (Figure 7B; Supplementary Figure S3). Nevertheless, the fatty acid (FA) profile presented some changes in response to different GBH concentrations (Figure 7C). While on one hand there was an increase in 16:1n-9 and 18:1 (oleic acid) at 250 µg·L^−1^ in comparison to the control, a decrease was observed in 18:0, at 250 µg·L^−1^ and 500 µg·L^−1^, and 18:2 (linoleic acid) at 250 µg·L^−1^ GBH.

### 3.6. Energy Balance

**Figure 8:**
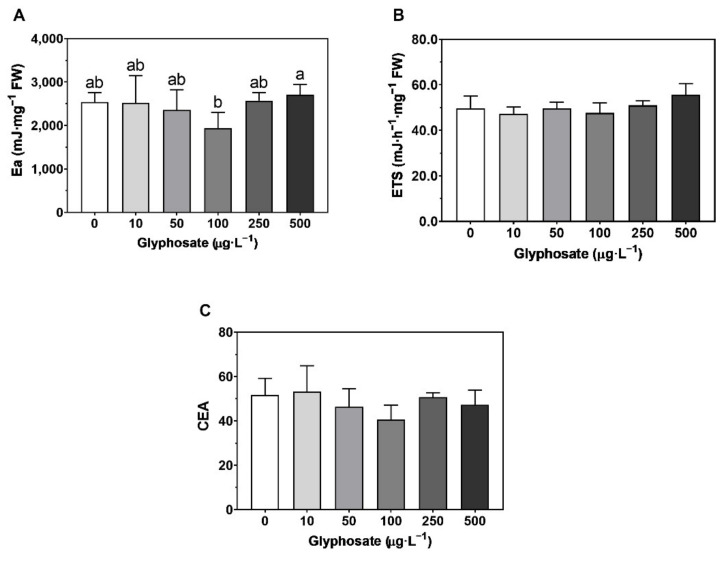
Energy balance: (**A**) energy available [Ea]; (**B**) energy consumption rate [ETS]; (**C**) cellular energy allocation [CEA]) in *Ulva lactuca* following a 48-h exposure to glyphosate-based herbicide formulation in different concentrations (mean ± s.d., *n* = 5, different letters indicate significant differences at *p* < 0.05).

**Figure 9:**
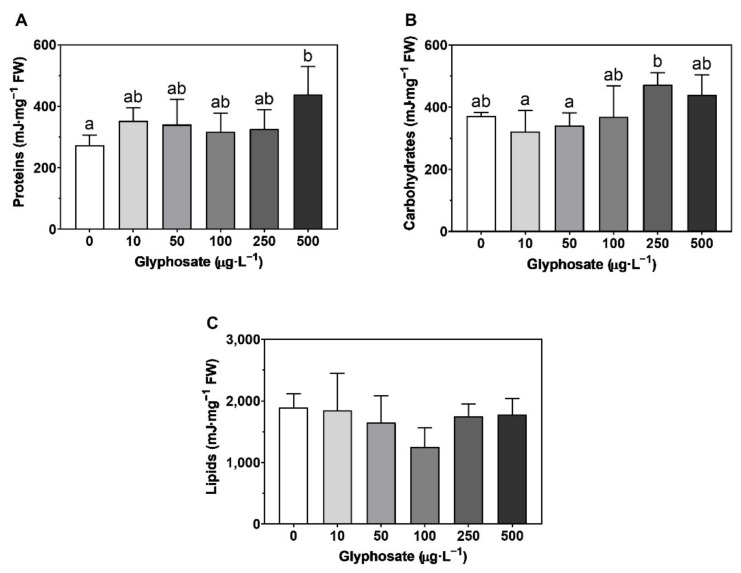
Energy availability: (**A**) carbohydrates; (**B**) proteins; (**C**) lipids in *Ulva lactuca* following a 48-h exposure to glyphosate-based herbicide formulation in different concentrations (mean ± s.d., *n* = 5, different letters indicate significant differences at *p* < 0.05).

Regarding the energy available in *U. lactuca*, there was only a significant decrease in the energy available after exposure to 100 µg·L^−1^ (Figure 8A), although no significant changes were observed in the energy consumption rate (Figure 8B) or the cellular energy allocation (Figure 8C) with the different GBH concentrations. Furthermore, a significant increase in proteins was observed after exposure to 500 µg·L^−1^ (Figure 9A), although no significant changes were observed in carbohydrates (Figure 9B) and lipids (Figure 9C) when compared to the control.

### 3.7. Classification Using OJIP and LIF Datasets

**Figure 10:**
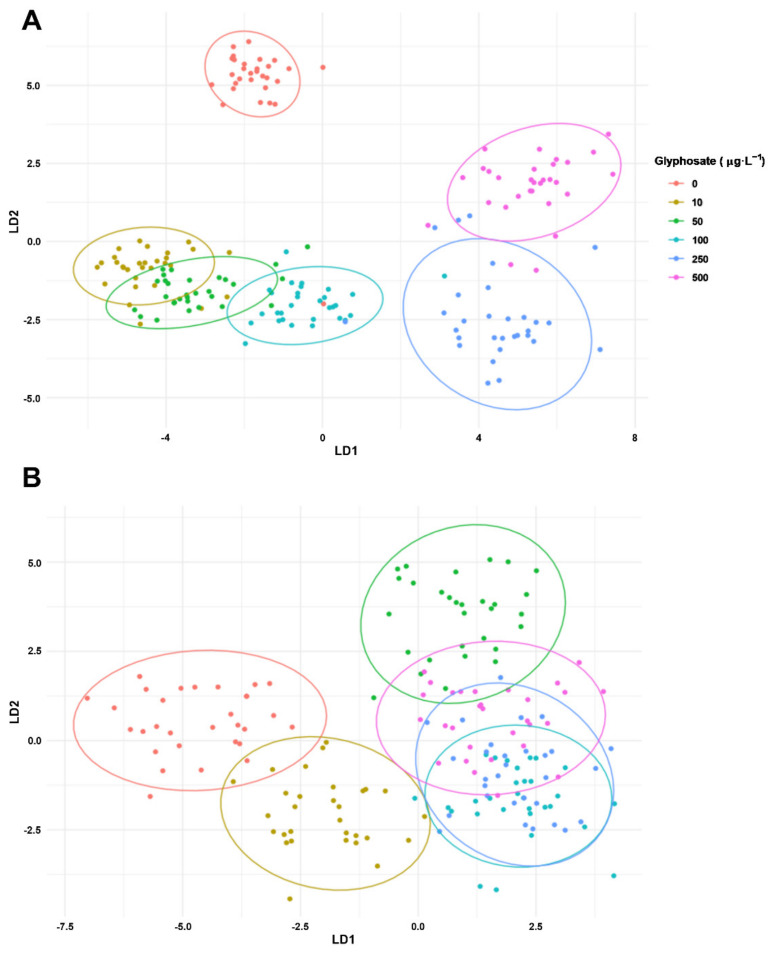
Linear discriminant analysis (LDA) using the (**A**) OJIP dataset and (**B**) the laser-induced fluorescence (LIF) dataset of *Ulva lactuca* following a 48-h glyphosate-based herbicide formulation in different concentrations. Ellipses group samples with lower statistical distance based on Euclidean resemblances.

The linear discriminant analysis using the Kautsky curve fluorescence dataset of *U. lactuca* disks exposed to different GBHs allowed a good separation of the control samples from the lowest GBH concentrations (10 to 100 µg·L^−1^) and the two highest concentrations (250 and 500 µg·L^−1^ GBH) (Figure 10A). Overall, the classification of the *U. lactuca* disks based on the Kautsky curves fluorescence showed a high accuracy of 86 % (Table S2).

On the other hand, the same analysis using the LIF fluorescence dataset of *U. lactuca* disks only allowed the separation of control samples from the lowest GBH (10 µg·L^−1^), with low classification accuracies across the 50 µg·L^−1^ to 500 µg·L^−1^ GBH (Figure 10B). This led to a lower overall classification accuracy of 69% (Table S3), mostly due to misclassifications in the control and 250 µg·L^−1^ GBH samples.

## 4. Discussion

The increased use of herbicides worldwide poses a continued threat to marine ecosystems. Glyphosate-based products not only can have a direct effect through chelation and, thus, deplete the bioavailability of macro- and micro-nutrients, such as calcium and magnesium, essential to growth [88] but also potentially have bottom-up effects in the trophic webs affecting both microalgae [13] and macroalgae [36].

The GBH had a clear effect on the profile of several photochemical variables of *U. lactuca*, particularly lower photosynthetic efficiency at higher concentrations. Although GBH induced a decrease in chlorophyll content [89] in the intermediate concentrations (10 to 100 µg·L^−1^), photosynthetic pigments recovered at the higher concentrations (250 and 500 µg·L^−1^), a mechanism that could compensate for the decrease in the energy fluxes due to lower efficiency of the reaction centers. Furthermore, the accumulation of antheraxanthin at the highest concentration may indicate a tipping point or threshold for this species to develop some tolerance to the herbicide. This increase in antheraxanthin was also observed in *Ulva pertusa* when subjected to desiccation [90] and *Ulva prolifera* after exposure to high light (900 µmol photons m^−2^ s^−1^) [91] reinforcing the role of this pigment in the protection of the thylakoid membrane against environmental stress. Many biological responses to the environment present non-linear relationships with their environment [92], giving the appearance of resilience to stress just before an ecological shift or threshold [93,94,95,96,97].

On the other hand, no changes were observed in the total fatty acid content, with 16:0 (palmitic acid) the predominant FA, typical in seaweeds [98]. Moreover, with increasing GBHs, total fatty acids presented a decreasing trend while the saturation degree increased. This can be the result of decreased activity of fatty acid desaturases [99], leading not only to a decrease in membrane fluidity, a common response to stress [100], but could also lead to the induction of defense responses [101]. Furthermore, the observed changes in the FA 16:1n-9, 18:1 and 18:2 are typically associated with plant defense signaling against pathogens [102]. While the accumulation of 16:1 is associated with increased resistance to pathogens [103,104], this expected increase was not observed in 18:2 [105], which decreased at 250 µg·L^−1^ GBH. Changes in 18:1 (oleic acid) are also related to defense signaling although its levels are very dynamic and sometimes contradictory, involving shuttles between the stroma and the nucleoid. Although decreases in oleic acid can induce the expression of resistance genes [106], increases in *U. lactuca* of this FA also boost defense mechanisms [107]. All these changes occur when *U. lactuca* is in its lower physiological fitness and, thus, at increased susceptibility to infections; nevertheless, these signals may allow the onset of mechanisms to protect the chloroplast and allowed to restore FA to control levels under the highest concentration, which, again, indicates a threshold induced response to environmental stress, as previously discussed.

However, the deleterious effect of GBHs on the chloroplastidial ETC of *U. lactuca* was observed at concentrations ranging between 50 and 500 µg·L^−1^, negatively affecting all energy fluxes (absorbed, trapped, transported, and dissipated), decreasing the contribution of the light reactions, the number of available reaction centers, and the pool of quinones, and an increase in the dysconnectivity between PSII. Nevertheless, this can also function as a regulatory mechanism to dissipate the excess energy [108], in conjunction with the previously mentioned accumulation of antheraxanthin at high GBH concentrations [90,91]. At the threshold concentration (250 µg·L^−1^ GBH), the macroalgae *U. lactuca* presented a decrease in the energy transduction at the ETC, from Q_A_^–^ to plastocyanin (ψ_0_/(1 − ψ_0_)), particularly from Q_A_ to plastoquinone since the energy transport from PQH_2_ to the PS I was not affected (RE_0_/RC). Therefore, the energy reaching the PS I was lower, which is further confirmed by a shift in the redox equilibrium between photosystems towards the PS II (ψ_E0_/(1 − ψ_E0_)), originating a decrease in photosynthetic effectiveness by impairment of both light and dark reactions of photosynthesis [13,54]. Therefore, GBH can interfere, not only via the shikimate pathway but also via the photosynthetic apparatus at the PSII and pool of quinones level, as observed in other primary producers [9,34,109,110].

Although only the highest GBH concentration led to a shift in the wavelength emission maxima, an increase in the well-established LIF parameter F_r_/F_fr_ was detected in concentrations ranging between 50 and 500 µg·L^−1^ GBH (except for 250 µg·L^−1^ GBH). These differences are related to the observed changes in photosynthetic and photoprotective pigments, fatty acid composition and other metabolic adaptations, as observed in other studies [56,57]. Although with some limitations in terms of GBH concentration resolution, this measurement has the potential to be a stress indicator, and laser-induced fluorescence is a tool with potential for plant stress detection [111,112] and should be explored in future studies.

GBHs induce oxidative stress in plants and the respective antioxidant defense system [9,113]. Although in *U. lactuca* no changes in CAT and GR were observed, an increase in APX activities was observed. On the other hand, a decreased activity of SOD at 250 µg·L^−1^ occurred which can lead to the accumulation of ROS, namely superoxide anion, having deleterious effects due to the oxidation of biological components, such as proteins and lipids [114,115]. The decreased activity of SOD will impair the superoxide anion dismutation into hydrogen peroxide and thus, even under high APX activities, the deleterious effects of this anion will still be felt in several cellular compartments [115], which can be part of the reason for poor fitness at this concentration.

Overall, the energy balance presented no significant changes either in the available energy (Ea), the energy consumption by the mitochondrial Electron Transport System (ETS) or the cellular energy allocation (CEA). Furthermore, *U. lactuca* showed no changes in carbohydrates and lipids, and only an increase in proteins at the highest concentration, which accumulated as a response to environmental stress [116,117]. This high resilience is not unexpected since *Ulva* spp. are associated with eutrophicated marine environments [118,119] and, once more, an indication of the induction of protective mechanisms following a contaminant exposure threshold.

After evaluation of the measured parameters, the linear discriminant analysis using the Kautsky fluorescence dataset turned out to be very efficient in the classification of the GBH contamination effect on *U. lactuca* photochemistry. This dataset allows the separation of the different treatments particularly with a good resolution at higher GBH concentrations. This technique has been increasingly shown to be a particularly valuable tool for ecological pollution assessment [13,53,54,55,120], presenting itself as a potential easy-to-access toxicity biomarker. Although the LIF measurements did not produce a good classification system, they still allowed discrimination between control and contaminated samples. Beyond the reinforced application of these non-invasive optical techniques for ecotoxicological assessment, the difference in accuracy observed in both fluorometric techniques also reflects the biological response of *U. lactuca*. Therefore, since LIF is more sensitive to changes directly occurring at the chlorophyll molecules and PSII levels [56], PAM analysis presented the highest classification efficiency of the samples exposed to different GBHs reinforcing the previously discussed findings that point to a more severe impact of GBHs on chloroplastidial ETC, particularly at the PSII and pool of quinones level [9,10,34,109].

Furthermore, since *U. lactuca* can induce tolerance to higher GBH concentrations (500 µg·L^−1^), it can lead to biomagnification of this pollutant, acting as a vector of contamination and bioaccumulation in the food web, including animal populations and, thus, indirectly also human populations. On the other hand, it could also be a potential bioremediation tool, increasing the rate of GBH photodegradation [52,121].

## 5. Conclusions

The present study indicates that GBHs have the potential to affect macroalgae, although, at the highest concentration, *U. lactuca* showed a resilience effect after reaching a tipping point, returning most photochemical and biochemical characteristics to the control values. This non-linear response may have a significant negative effect on the trophic web as the herbicide may bioaccumulate once the tolerance mechanisms are induced. The application of the non-invasive optical techniques PAM and LIF could provide better monitoring of such impacts and allow improved management of coastal aquatic habitats.

## Figures and Tables

**Table 1:** The wavelength at maxima fluorescence in the red and far-red regions, and red/far-red fluorescence ratios (F680/F735) in *Ulva lactuca* following a 48-h exposure to a glyphosate-based herbicide formulation in different concentrations (mean ± s.d., *n* = 30, different letters indicate significant differences between treatments at *p* < 0.05; numbers in bold indicate significant deviation from control).

Glyphosate Concentration (µg·L^−1^)	Wavelength in Maxima Fluorescence (Red Region)	Wavelength in Maxima Fluorescence (Far-Red Region)	Red/Far-Red Fluorescence Ratio (F680/F735)
0	681.06 ± 0.35 ^ab^	731.29 ± 9.88	41.12 ±18.35 ^a^
10	680.97 ± 0.24 ^ab^	732.94 ± 8.78	37.25 ± 16.01 ^a^
50	680.88 ± 0.28 ^a^	730.46 ± 8.62	**103.63 ± 58.18** **^b^**
100	681.16 ± 0.28 ^bc^	733.90 ± 11.32	**73.45 ± 33.40 ^bc^**
250	681.09 ± 0.23 ^bc^	733.73 ± 8.56	53.78 ± 25.43 ^ac^
500	**681.32 ± 0.29 ^c^**	732.42 ± 8.11	**70.80 ± 47.18 ^bc^**

**Table 2:** Pigment profile content (µg·g^−1^ FW) in *Ulva lactuca* following a 48-h exposure to a glyphosate-based herbicide formulation in different concentrations (mean ± s.d., *n* = 3, different letters indicate significant differences between treatments at *p* < 0.05; numbers in bold indicate significant deviation from control).

	Glyphosate Concentration (µg·L^−1^)					
	0	10	50	100	250	500
Chlorophyll *a*	56.03 ± 8.93 ^a^	**15.80 ± 4.19 ^b^**	**18.00 ± 6.06 ^b^**	**14.40 ± 4.66 ^b^**	32.23 ± 2.89 ^ab^	55.53 ± 27.19 ^a^
Chlorophyll *b*	38.90 ± 7.95 ^a^	**10.31 ± 2.76 ^cd^**	**12.05 ± 3.93 ^bcd^**	**8.64 ± 2.54 ^d^**	20.27 ± 2.66 ^abc^	31.20 ± 17.59 ^ab^
Pheophytin *a*	2.12 ± 1.83	0.25 ± 0.24	0.46 ± 0.41	0.10 ± 0.02	0.96 ± 0.62	0.42 ± 0.09
Pheophytin *b*	7.32 × 10^−10^ ± 6.07 × 10^−10 ab^	5.96 × 10^−10^ ± 6.09 × 10^−10 b^	3.23 × 10^−10^ ± 4.97 × 10^−11 b^	0.06 ± 0.11 ^ab^	6.43 × 10^−10^ ± 3.26 × 10^−10 ab^	0.9 ± 0.6 ^a^
Lutein	3.02 ± 1.03 ^ab^	**0.59 ± 0.15 ^c^**	0.82 ± 0.23 ^abc^	0.70 ± 0.27 ^bc^	2.52 ± 1.47^abc^	4.04 ± 1.92 ^a^
β-Carotene	1.32 ± 0.22 ^ab^	0.40 ± 0.22 ^ab^	0.53 ± 0.21 ^ab^	0.52 ± 0.20 ^ab^	0.57 ± 0.67 ^b^	2.06 ± 0.42 ^a^
Zeaxanthin	1.40 ± 0.24 ^ab^	0.55 ± 0.29 ^ab^	0.56 ± 0.22 ^ab^	0.55 ± 0.21 ^ab^	0.60 ± 0.71 ^b^	2.43 ± 0.36 ^a^
Antheraxanthin	2.97 × 10^−11^ ± 1.52 × 10^−11 a^	0.13 ± 0.22 ^a^	0.49 ± 0.21 ^ab^	0.76 ± 0.61 ^ab^	0.72 ± 1.24 ^ab^	**3.81 ± 0.34 ^b^**
Violaxanthin	0.80 ± 0.71	0.44 ± 0.20	0.34 ± 0.28	0.13 ± 0.11	0.03 ± 0.05	0.18 ± 0.31
Auroxanthin	4.98 ± 1.70 ^ab^	1.93 ± 0.83 ^ab^	1.81 ± 0.77 ^ab^	1.59 ± 0.54 ^ab^	1.52 ± 2.49 ^b^	6.11 ± 1.60 ^a^

## Data Availability

Not applicable.

## Supplementary Materials

The following supporting information can be downloaded at: https://www.mdpi.com/article/10.3390/toxics10080430/s1, Figure S1: Rapid light curves and derived parameters; Figure S2: Lipid peroxidation quantification; Figure S3: Major fatty acids classes; Table S1: Fluorometric analysis parameters and their description; Table S2: Confusion matrix for LDA (Kautsky); Table S3: Confusion matrix for LDA (LIF).
