# Supplementary material for: Effects of Glyphosate-Based Herbicide on Primary Production and Physiological Fitness of the Macroalgae Ulva lactuca
Source: Toxics. 2022 Jul 28;10(8):430. doi: 10.3390/toxics10080430 (PMC9415031; doi:10.3390/toxics10080430)
Supplement: Supplementary file 1 [file toxics-10-00430-s001.zip › toxics-1831106-supplementary.pdf]

toxics MDPI

Supplementary Materials: Effects of Glyphosate-Based Herbicide on Primary Production and Physiological Fitness of the Macroalgae *Ulva lactuca*

Ricardo Cruz de Carvalho, Eduardo Feijão, Ana Rita Matos, Maria Teresa Cabrita, Andrei B. Utkin, Sara C. Novais, Marco F. L. Lemos, Isabel Caçador, João Carlos Marques, Patrick Reis-Santos, Vanessa F. Fonseca and Bernardo Duarte

Table S1. Fluorometric analysis parameters and their description.

OJIP-test	
Area	Corresponds to the oxidized quinone pool size available for reduction and is a function of the area above the Kautsky plot
N	Reaction centre turnover rate
S_M	Corresponds to the energy needed to close all reaction centres
S_S	Corresponds to the smallest possible normalized total area when each QA is reduced only once (single turnover)
M_0	Net rate of PS II RC closure
γ_{RC}	Probability that a PSII chlorophyll molecule function as a RC
P_G	Grouping probability between the two PSII units
ABS/CS	Absorbed energy flux per cross-section
TR/CS	Trapped energy flux per cross-section
ET/CS	Electron transport energy flux per cross-section
DI/CS	Dissipated energy flux per cross-section
RC/CS	Number of available reaction centres per cross-section
TR_0/DI_0	Contribution or partial performance due to the light reactions for primary photochemistry
δ_{R0} / $(1-\delta_{R0})$	Contribution of PSI, reducing its end acceptors
ψ_0 / $(1-\psi_0)$	Contribution of the dark reactions from QA− to PC
ψ_{E0} / $(1-\psi_{E0})$	Equilibrium constant for the redox reactions between PS II and PS I
RE_0/RC	Electron transport from PQH_2 to the reduction of PS I end electron acceptors
RC/ABS	Reaction centre II density within the antenna chlorophyll bed of PS II

Table S2. Confusion matrix for linear discriminant analysis (LDA) performed for the Kautsky curve fluorescence dataset in *Ulva lactuca* following a 48-h exposure to a glyphosate-based herbicide (GBH) formulation in different concentrations.

	Kautsky	Predicted						Class	Overall
	GBH (μg L^{-1})	0	10	50	100	250	500	accuracy (%)	accuracy (%)
Observed	0	29	0	0	1	0	2	91	86
	10	0	25	6	1	0	0	78	
	50	0	2	23	2	0	0	85	
	100	0	2	1	25	0	0	89	
	250	0	0	0	0	27	2	93	
	500	1	1	0	1	3	26	81	

Table S3. Confusion matrix for linear discriminant analysis (LDA) performed for the laser-induced fluorescence (LIF) dataset in *Ulva lactuca* following a 48-h exposure to a glyphosate-based herbicide (GBH) formulation in different concentrations.

	LIF	Predicted						Class	Overall
	GBH (μg L^{-1})	0	10	50	100	250	500	accuracy (%)	accuracy (%)
Observed	0	17	3	1	1	1	2	68	69
	10	3	20	1	1	0	3	71	
	50	2	2	23	1	3	2	70	
	100	2	1	3	22	1	2	71	
	250	3	3	2	4	23	2	62	
	500	3	1	0	1	2	19	73	

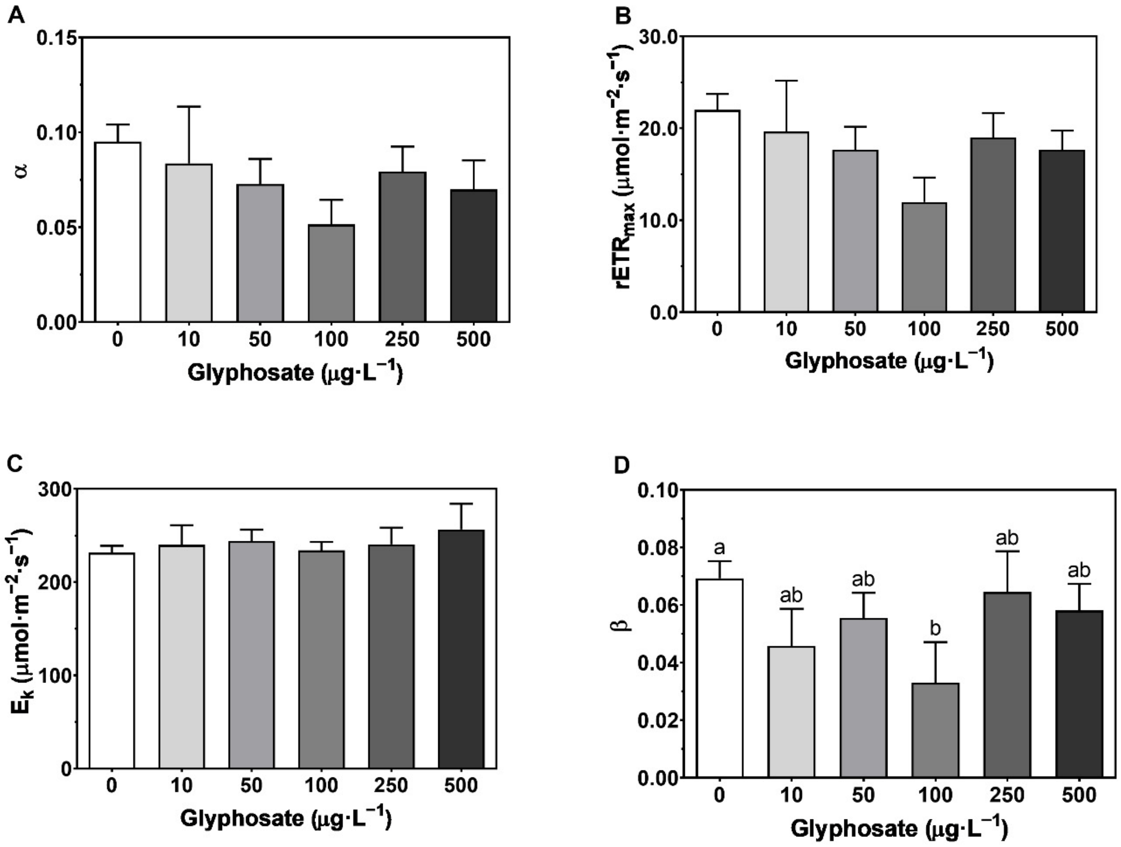

Figure S1. Rapid light curves and derived parameters (**A**, photosynthetic efficiency (α); **B**, relative maximum electron transport rate ($rETR_{max}$); **C**, light saturation (E_k); **D**, photoinhibition (β)) in *Ulva lactuca* following a 48-h exposure to a glyphosate-based herbicide formulation in different concentrations (mean ± s.d., n = 3, different letters indicate significant differences at $p < 0.05$).

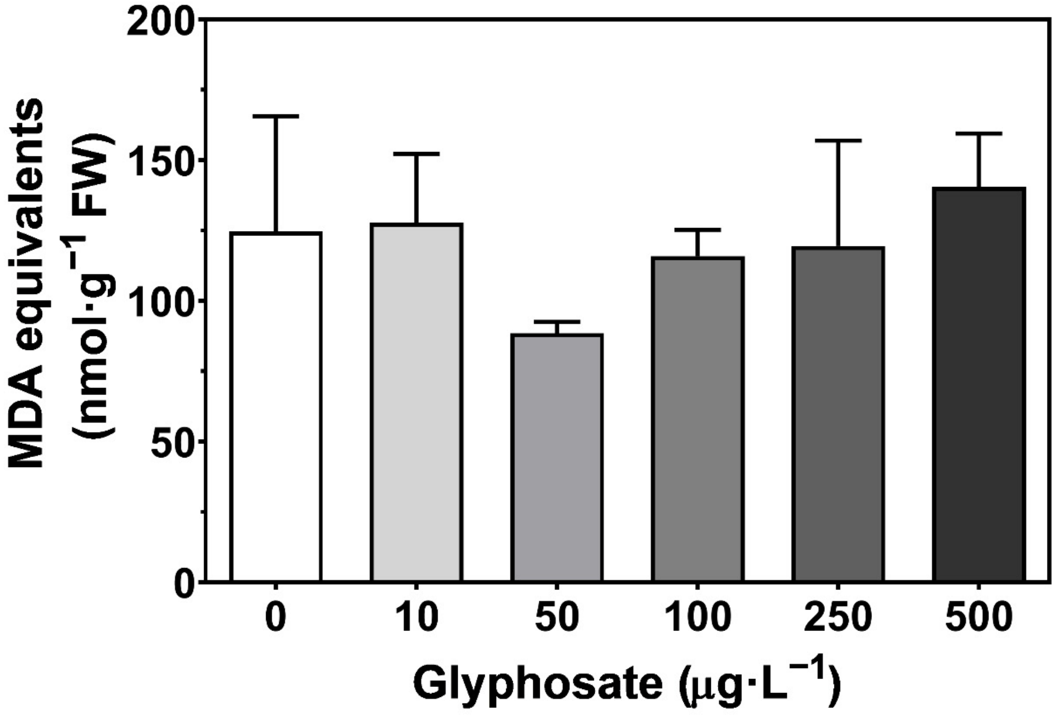

Figure S2. Lipid peroxidation quantification, measured as malondialdehyde (MDA) equivalents, in *Ulva lactuca* following a 48-h exposure to a glyphosate-based herbicide formulation in different concentrations (mean ± s.d., n = 5).

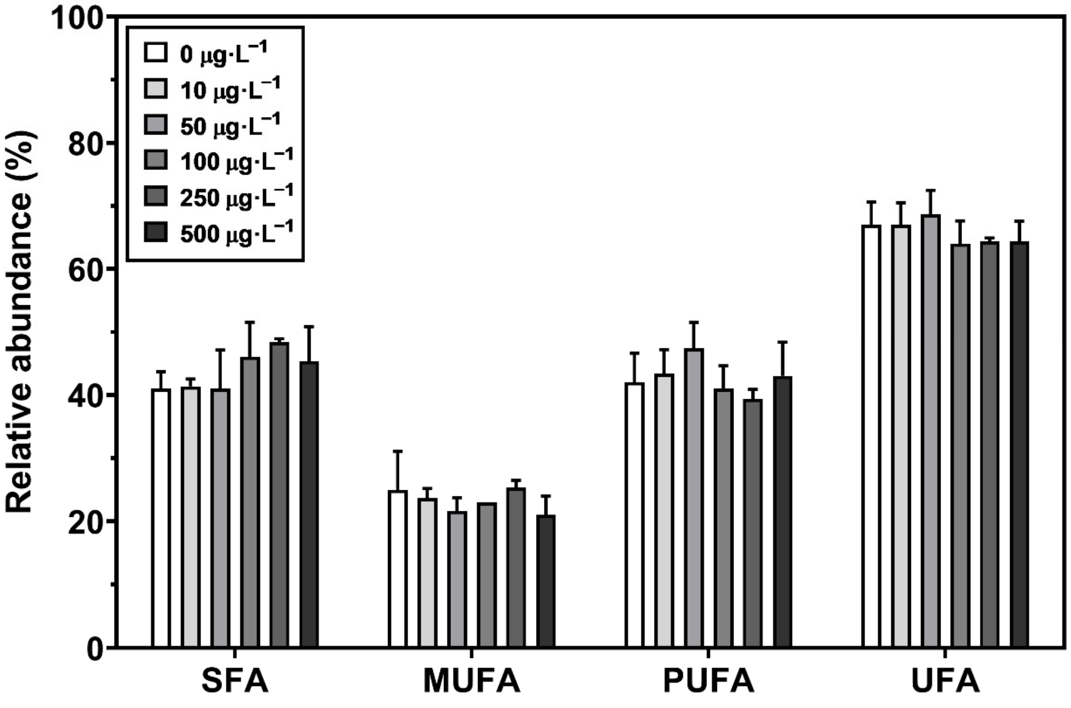

Figure S3. Major fatty acids classes (saturated fatty acids (SFA), monounsaturated fatty acids (MUFA), polyunsaturated fatty acids (PUFA) and unsaturated fatty acids (UFA) relative abundance) in *Ulva lactuca* following a 48-h exposure to a glyphosate-based herbicide formulation in different concentrations (mean ± s.d., n = 5).
